# Supplementary material for: Adsorptive Behavior of Cu2+ and Benzene in Single and Binary Solutions onto Alginate Composite Hydrogel Beads Containing Pitch Pine-Based Biochar
Source: Polymers (Basel). 2022 Aug 25;14(17):3468. doi: 10.3390/polym14173468 (PMC9460076; doi:10.3390/polym14173468)
Supplement: Supplementary file 1 [file polymers-14-03468-s001.zip › polymers-1883007-supplementary.pdf]

Adsorptive behavior of Cu^{2+} and benzene in single and binary solutions onto alginate composite hydrogel beads containing pitch pine-based biochar

Saerom Park[a,†], Jeong Woo Lee[a,†], Ji Eun Kim[b], Gwangnam Kang[b], Hyung Joo Kim[a], Yong-Keun Choi[a,*], Sang Hyun Lee[a,*]

[a] Department of Biological Engineering, Konkuk University, Seoul 05029, Republic of Korea

[b] R&D Center, ATE Corporation, Seoul 05029, Republic of Korea

*** Corresponding authors**

Yong-Keun Choi

Tel.: +82-2-2049-6111

E-mail: dragonrt@konkuk.ac.kr

Sang Hyun Lee

Tel.: +82-2-2049-6269

E-mail: sanghlee@konkuk.ac.kr

[†]Saerom Park and Jeong Woo Lee are equally contributed to this study.

Table S1. Equations and parameters of the adsorption kinetics and isotherm models

Adsorption models	Equations	Parameters	ref.
Kinetics			
Pseudo-first-order	$\ln(q_e - q_t) = lnq_{e1} - k_1 t$	q_{e1}: calculated adsorption capacity at equilibrium<br>k_1: rate constant	
Pseudo-second-order	$\frac{t}{q_t} = \frac{1}{k_2 q_{e2}^2} + \frac{t}{q_{e2}}$	q_{e2}: calculated adsorption capacity at equilibrium<br>k_2: rate constant<br>h ($k_2 q_{e2}^2$): initial adsorption rate	
Elovich	$q_t = \frac{1}{\beta}\ln(\alpha\beta) + \frac{1}{\beta}lnt$	α: initial adsorption rate<br>β: desorption constant	[43], [53]
Intraparticle diffusion	$q_t = k_{id}t^{0.5} + C$	k_{id}: rate constant<br>C: constant	
Liquid film diffusion	$\ln\left(1 - \frac{q_t}{q_e}\right) = -k_{fd}t + C$	k_{fd}: rate constant<br>C: constant	
Isotherms			
Langmuir	$\frac{C_e}{q_e} = \frac{C_e}{q_m} + \frac{1}{q_m k_L}$<br>$R_L = \frac{1}{1 + k_L C_0}$	q_m: maximum adsorption capacity<br>k_L: Langmuir constant<br>R_L: separation factor	
Freundlich	$logq_e = logk_F + \frac{1}{n}logC_e$	k_F: Freundlich constant<br>n: heterogeneity factor	
Dubinin–Radushkevich	$lnq_e = lnq_m - k_{DR}\varepsilon^2$<br>$\varepsilon = RTln(1 + \frac{1}{C_e})$<br>$E = \frac{1}{\sqrt{-2k_{DR}}}$	q_m: maximum adsorption capacity<br>k_{DR}: Dubinin–Radushkevich constant<br>E: mean energy of adsorption	[43], [53]
Elovich	$\ln\left(\frac{q_e}{C_e}\right) = lnk_E q_m - \frac{q_e}{q_m}$	q_m: maximum adsorption capacity<br>k_E: Elovich constant	

Table S2. Yield and BET specific surface area of pitch pine-based biochars produced at different pyrolytic temperatures (350, 550, and 750 °C)

Pyrolysis temperature (ºC)	Yield (%)	BET surface area (m^2/g)
350	35.6	1.7
550	20.1	297.4
750	17.0	6.7

Table S3. Physical properties of the alginate and alginate/biochar hydrogel beads

Added biochar content in 4% alginate solution (%)	Dried bead size (mm)*	Dry weight (mg)*	Measured biochar content in alginate/biochar beads (mg)
0	1.2	0.7	-
1	1.6	1.1	0.27
4	2.2	2.1	1.21
10	2.7	3.8	3.24

* Measured after drying at 60 °C for 24 h

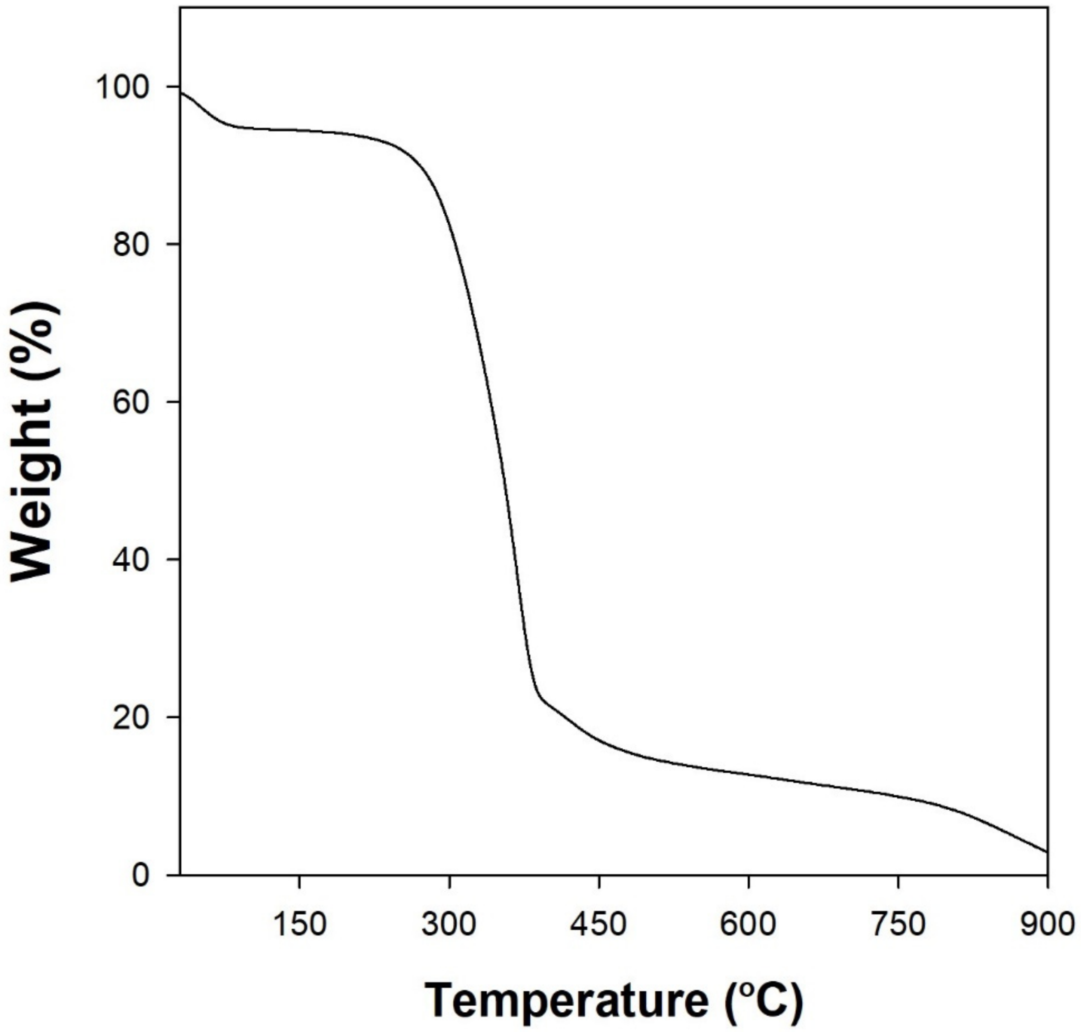

Figure S1. TGA thermogram of pitch pine powder.

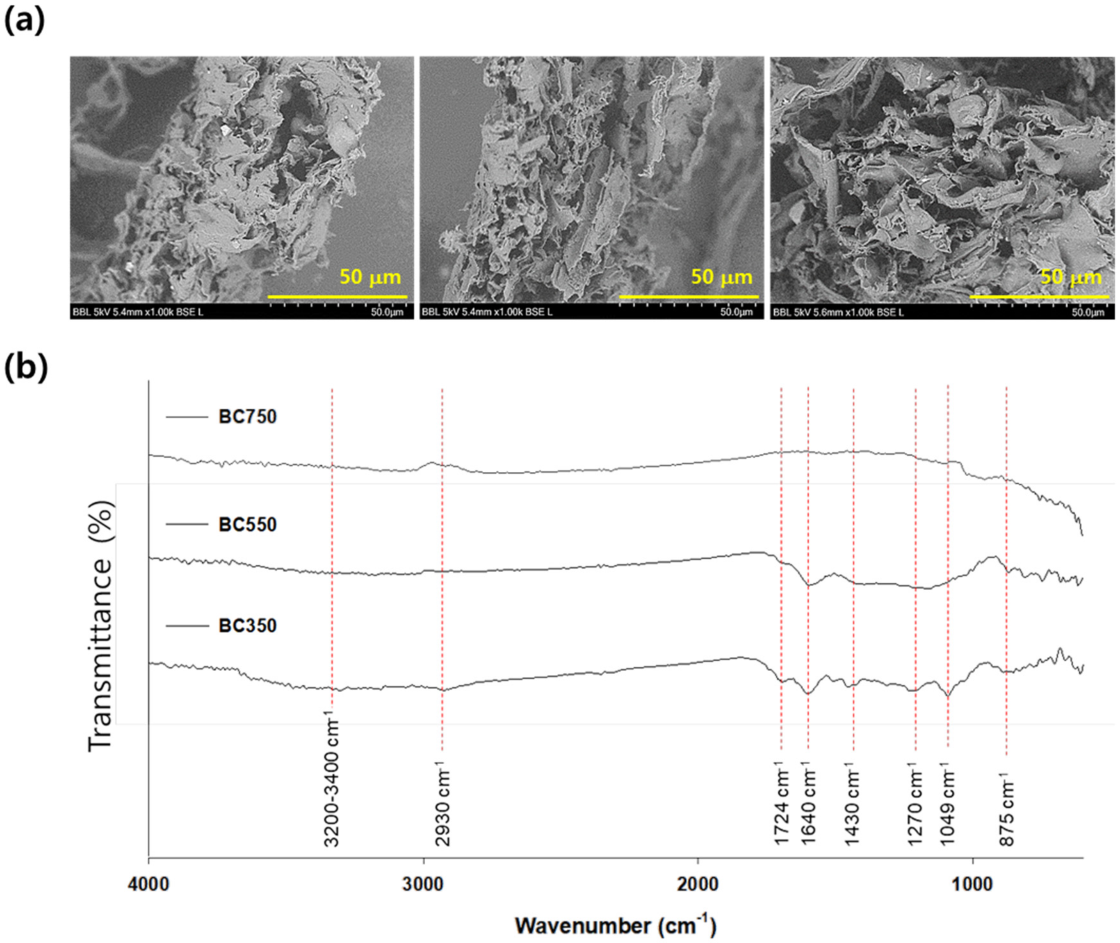

Figure S2. SEM images (a) and FTIR spectra (b) of biochars (BC350, BC550, and BC750) obtained at 350, 550, and 750 °C, respectively.

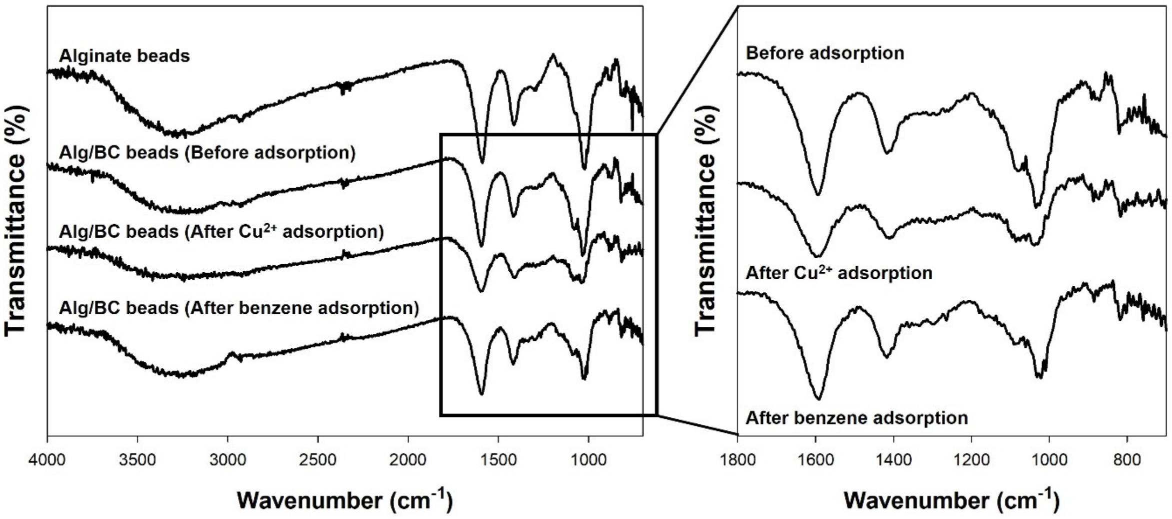

Figure S3. FTIR spectra of alginate and alginate/biochar hydrogel beads (prepared with 4% alginate and 4% BC) before and after Cu^{2+} and benzene adsorption.

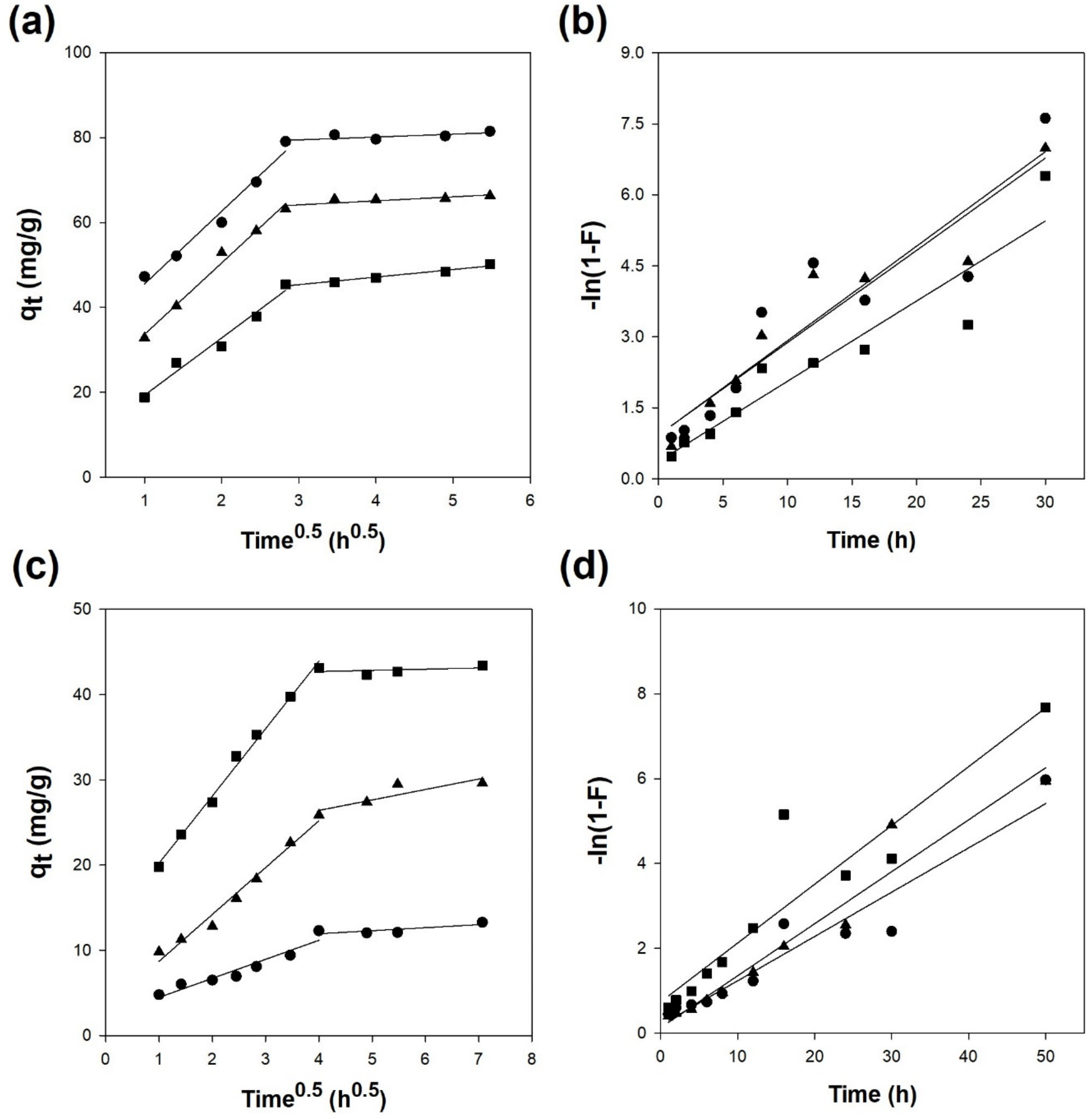

Figure S4. Diffusion kinetics studies of Cu^{2+} (a and b), and benzene (c and d) adsorption onto alginate and alginate/biochar hydrogel beads (●: 4% alginate / 0% biochar; ▲: 4% alginate / 1% biochar; ■: 4% alginate / 4% biochar). Plots of intraparticle diffusion model (a and c) and liquid film diffusion model (b) and (d).

43. Gürkan, E.H.; İlyas, B.; Tibet, Y. Adsorption performance of heavy metal ions from aqueous solutions by a waste biomass based hydrogel: Comparison of isotherm and kinetic models. Int. J. Environ. Anal. Chem. 2021, 1–18.

53. Oyelude, E.O.; Awudza, J.A.; Twumasi, S.K. Equilibrium, kinetic and thermodynamic study of removal of eosin yellow from aqueous solution using teak leaf litter powder. Sci. Rep. 2017, 7, 1–10.
